# A novel approach to craniofacial analysis using automated 3D landmarking of the skull

**DOI:** 10.1038/s41598-024-63137-1

**Published:** 2024-05-29

**Authors:** Franziska Wilke, Harold Matthews, Noah Herrick, Nichole Dopkins, Peter Claes, Susan Walsh

**Affiliations:** 1 Department of Biology, Indiana University Indianapolis, Indianapolis, IN USA; 2 Department of Human Genetics, KU Leuven, Leuven, Belgium; 3 Murdoch Children’s Research Institute, Melbourne, VIC Australia; 4 Medical Imaging Research Center, University Hospitals Leuven, Leuven, Belgium; 5 Department of Electrical Engineering, ESAT/PSI, KU Leuven, Leuven, Belgium

**Keywords:** Image processing, Molecular imaging

## Abstract

Automatic dense 3D surface registration is a powerful technique for comprehensive 3D shape analysis that has found a successful application in human craniofacial morphology research, particularly within the mandibular and cranial vault regions. However, a notable gap exists when exploring the frontal aspect of the human skull, largely due to the intricate and unique nature of its cranial anatomy. To better examine this region, this study introduces a simplified single-surface craniofacial bone mask comprising of 6707 quasi-landmarks, which can aid in the classification and quantification of variation over human facial bone surfaces. Automatic craniofacial bone phenotyping was conducted on a dataset of 31 skull scans obtained through cone-beam computed tomography (CBCT) imaging. The MeshMonk framework facilitated the non-rigid alignment of the constructed craniofacial bone mask with each individual target mesh. To gauge the accuracy and reliability of this automated process, 20 anatomical facial landmarks were manually placed three times by three independent observers on the same set of images. Intra- and inter-observer error assessments were performed using root mean square (RMS) distances, revealing consistently low scores. Subsequently, the corresponding automatic landmarks were computed and juxtaposed with the manually placed landmarks. The average Euclidean distance between these two landmark sets was 1.5 mm, while centroid sizes exhibited noteworthy similarity. Intraclass coefficients (ICC) demonstrated a high level of concordance (> 0.988), with automatic landmarking showing significantly lower errors and variation. These results underscore the utility of this newly developed single-surface craniofacial bone mask, in conjunction with the MeshMonk framework, as a highly accurate and reliable method for automated phenotyping of the facial region of human skulls from CBCT and CT imagery. This craniofacial template bone mask expansion of the MeshMonk toolbox not only enhances our capacity to study craniofacial bone variation but also holds significant potential for shedding light on the genetic, developmental, and evolutionary underpinnings of the overall human craniofacial structure.

## Introduction

The field of phenomics—understanding the qualitative and quantitative traits that characterize a phenotype, is a fast-developing field^1^. Over the past two decades, numerous publications have not only unveiled genetic variants associated with phenotypes, but also made significant advancements in phenotyping methodologies^2,3^. Moreover, the emergence of new technologies has enabled us to capture high quality 3D scans, encompassing both hard and soft tissue structures^1,4,5^. Although there have been significant strides made in understanding facial soft tissue variation, with technical advances implemented for genome wide association studies (GWAS) on facial shape^3,6^, the underlying craniofacial structure remains largely unexplored. This is in part due to the intricate nature of the entire skull shape and challenges in acquiring large numbers of 3D scans. Nevertheless, understanding human craniofacial structure is pivotal due to its substantial contribution to our facial appearance, particularly owing to its relative independence from biological factors such as weight, and reduced susceptibility to age-related changes after reaching adulthood^7,8^. Hence, a comprehensive exploration of skull morphology is essential for gaining a holistic understanding of the genetic determinants governing human facial shape. Although a recent GWAS was performed on the cranial vault^9^, a more comprehensive study of the viscerocranium (craniofacial bone structure) is imperative to tie in with facial soft tissue research that has been so successful in recent years.

Typically, studies describing the shape of the human skull have predominantly been within the field of Anthropology. In this context, the shape of the skull has been used to categorize an individual’s sex and ancestral origins^10,11^. While sex often relies on visual indicators, the assessment of ancestry is more complex. Computational tools such as FORDISC^12^ utilize skull measurements to estimate ancestry but cannot account for admixture and smaller subpopulations. Furthermore, estimations of soft-tissue thickness have been employed for facial reconstruction from a skull^13,14^, and skull shape analyses provide insights on primates to *Homo sapiens* evolutionary processes^15,16^. In the medical realm, skull shape is often used to describe specific pathologies or act as a non-syndromic reference^17,18^. More recently, the dental and plastic surgery fields have also capitalized on skull shape analyses to aid in reconstructive surgical procedures and preoperative surgical planning. This can be accomplished by visualizing not only the shape differences between syndromic and non-syndromic skulls useful for diagnoses, but also representations of the potential outcome in surgical procedures^19–21^. However, many of these previous approaches were constrained by their reliance on manual cranial landmarks (usually less than 50) and the use of physical skulls or radiographs as data sources^22,23^. These conventional approaches bring inherent challenges. Firstly, the process is time-consuming, requires trained observers, and is prone to intra- and inter-observer error, thereby complicating standardization^24–26^. While shape analysis can be performed by considering the overall configuration of a few landmarks, such an approach trivializes the complete complexity of cranial facial shape^27,28^. Although, algorithms were developed in the early 2000s that have allowed automatic 3D dense phenotyping^29^, they have vastly improved since then^27,28,30^.

A more recent framework for automatic 3D dense phenotyping, “MeshMonk”, was introduced by White et al.^28^ in 2019. MeshMonk provides a facial soft tissue mesh comprising approximately 7160 points, accompanied by algorithms to facilitate the alignment of this mask to 3D facial scans. This framework has contributed a straightforward, standardized, and validated method to describe the phenotypic variation found in facial shape using large datasets by simplifying automated landmarking. While its application has led to multiple publications exploring the genetic architecture of the human face^2,3,6^, it is crucial to note however, that the framework does not include a hard-tissue component. Global registration masks using MeshMonk have been developed and utilized for specific segments of the skull, including the lower jaw^31^ and the cranial vault^9^, however, assessment of the facial bones within the craniofacial complex has not been explored.

To perform large-scale GWAS that explore hard structures of the human face, these requires a large number of bone scans to be processed accurately and efficiently. Fortunately, advancements in medical technology have increased the availability of these scans via Magnetic Resonance Imaging (MRI) and Computed Tomography (CT). The use of Cone Beam CT (CBCT) has also emerged as a prominent imaging technique in the dental field. CBCT scans have the advantage of lower radiation dosages than conventional CT scans and reduced costs, rendering them viable for research purposes. To facilitate and cover the broad range of bone scans available, a suitable landmarking approach must be devised to simplify the intricate aspects of skull morphology without sacrificing critical information, therefore ensuring a more comprehensive and powerful analysis.

This research aimed to develop a skull template mask and workflow for automated skull landmarking that increases the number of quasi-landmarks, whilst reducing placement error. Our mask extends the MeshMonk framework, enabling its application to both CT and CBCT skull scans, empowering researchers to analyze craniofacial bone structure more easily. This addition broadens the potential scope of investigations in phenomics, medical, and dental research, facilitating a comprehensive exploration of the genetic determinants underlying human skull formation, particularly craniofacial bone morphology.

## Materials and methods

### Participant recruitment and study sample

Participants for this research were collected at Indiana University Indianapolis (IUI). The study underwent ethical review and received approval from the institutional review board (IU IRB 1801992304) of Indiana University (Federal-wide Assurance Number FWA00003544) that supports compliance with federal regulations for the protection of human subjects in research. Prior to participating, individuals provided informed consent, which included an acknowledgement of potential radiation exposure associated with Cone Beam Computed Tomography (CBCT) imaging. To ensure anonymity, each participant was assigned a unique identification number, and all collected data were securely stored on a server accessible only to those with prior IRB approval. The study exclusively enrolled individuals aged 18 and above, excluding those with a history of significant facial trauma, individuals with incomplete data, or scans that lacked complete orbital information. In total, the dataset comprised 31 skulls.

CBCT imaging procedures were conducted at the IU School of Dentistry within the Orthodontics and Oral Facial Genetics Department, utilizing a Carestream 9300 machine manufactured by Carestream Health, Inc. (NY). All full-face scans adhered to specific parameters, including a field of view (FOV) of 17 cm × 13.5 cm (this encompasses all facial bones, whilst excluding most of the frontal bone/forehead), an X-ray tube current of 15 mA, an X-ray tube voltage of 90 kV, and a scan duration of 28 s. The scans themselves were administered by a qualified and licensed professional.

### DICOM extraction and data cleaning

The DICOM images obtained from the CBCT were processed in the free software 3D Slicer^32^. A threshold of between 400–600 to maximum Hounsfield units was set. The resulting mesh was filtered for the largest island to remove pieces of the spine and loose internal structures. In addition, minor holes were closed using the “closing smoothing” setting in 3D Slicer with a kernel size of 2.0 mm. The skull meshes were then imported into Blender^33^ where a half-cylindrical mesh was placed around the skull and the shrink-wrap modifier applied (Supplementary Fig. S1). In addition, the subdivision surface modifier was applied to increase the resolution. This process was repeated a further 5× with two decimate modifiers (un-subdivide setting) in-between to prevent the resulting mesh file from being too large. The output was a high polygon count mesh with an uneven vertex distribution. To counteract this, the meshes were reduced to 30,000 triangles in Meshmixer^34^ then evenly re-meshed, resulting in regularly spaced vertices, and a similar number of faces between meshes.

### Phenotyping

One skull mesh, chosen by the author as the skull with the highest quality due to is relative symmetry, least artifacts, and perceived normal range phenotype, was used as a preliminary mask and symmetrized in Blender (one half delete, the center vertices moved to X = 0 and the mesh mirrored) and re-meshed for an even distribution of vertices resulting in 9999 quasi-landmarks. A subset of 20 skulls were masked with this preliminary mask using the MeshMonk framework^28^ in Matlab^35^. This toolbox uses a 3-step process to non-rigidly align the mask to each target shape: (1) initialization is performed by placing eleven manual landmarks (Supplementary Fig. S2) (custom script within MeVisLab (available: http://www.mevislab.de/)) on both the mask and target shapes which are utilized to estimate the rigid registration, (2) rigid registration is optimized via iterative closest point registration, (3) non-rigid registration is performed to adapt the shape of the mask to the shape of the target mesh. MeshMonk settings^36^ were modified for the best result, and tested with a script to produce a type of ‘quality control’ test image that depicts the mask overlaying the original mesh (see Supplementary File). This resulted in all 20 skulls consisting of 9999 quasi-landmarks in corresponding anatomical locations. After Procrustes Superimposition for alignment and scaling, the skulls were averaged resulting in the final average mask. This mask was consequently symmetrized by averaging the original and its reflection.

The full set of 31 skulls were then registered via MeshMonk using the final average mask and eleven initial landmarks as required by MeshMonk. This process was repeated three times with three different sets of initial landmarks to test the reliability of the automatic landmarking using the newly developed bone mesh. The process from DICOM data to masked skull took approximately 30 min per skull, however this time depends predominantly on the speed of the CPU.

### Validation landmarks

**Figure 1:**
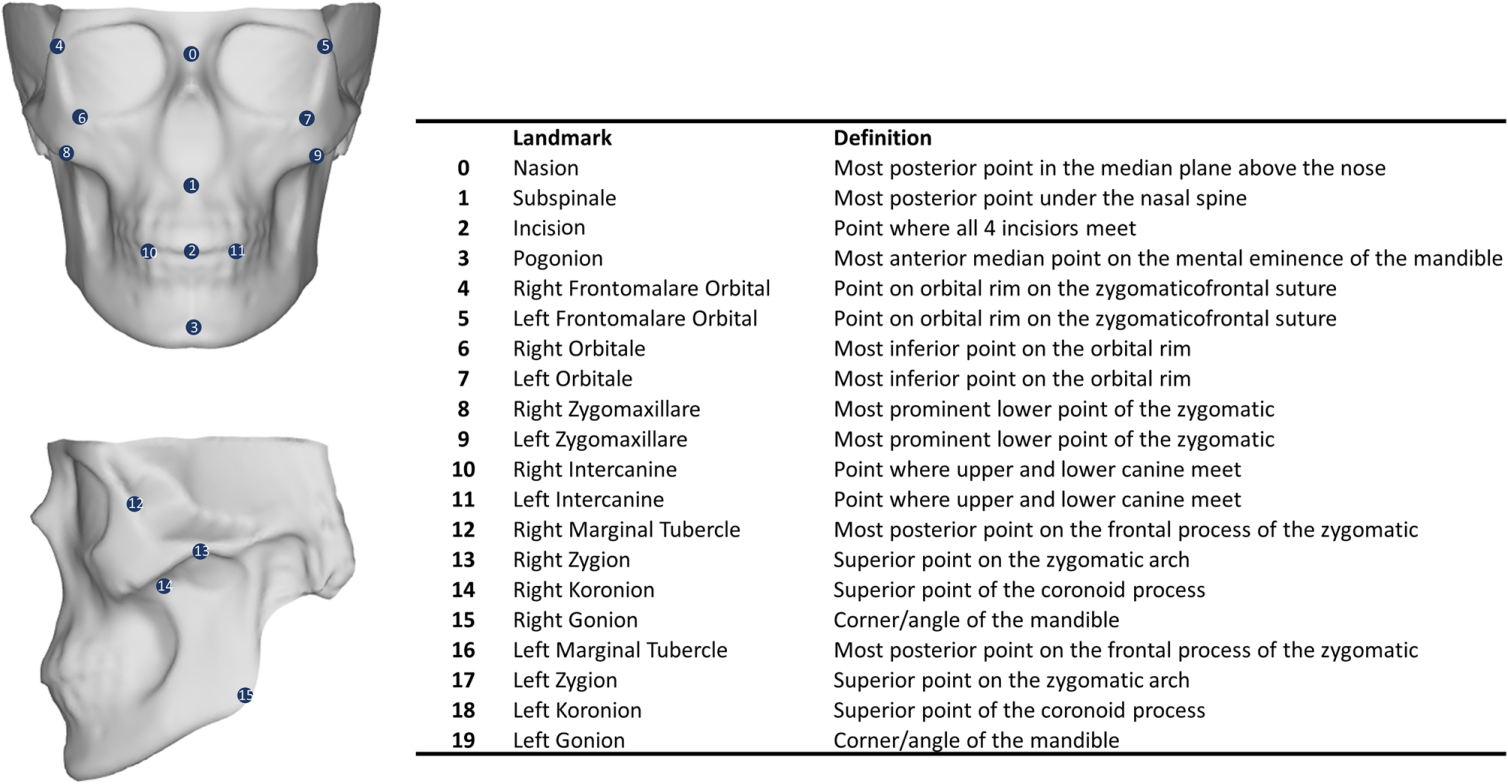
Overview of the manually placed landmarks. Landmark definitions were taken from^37,38^.

Validation, reliability, and accuracy assessments of the quasi-landmark placement via MeshMonk were conducted by comparing automatically placed landmarks with manually positioned landmarks. To assess this, the 31 skulls were landmarked manually with 20 craniofacial landmarks by three observers (Fig. 1). Observer 1 was well versed in this procedure due to their anthropological background, observer 2 had some landmarking experience from previous dental and facial studies, while observer 3 was completely untrained. Landmarks were chosen which evenly covered the skull and represented the areas consistently captured by CBCT with clear definitions taken from literature^37,38^. These “gold-standard” landmarks were placed on the skulls after shrink-wrapping and remeshing as described previously for a more accurate comparison. Each observer landmarked all 31 skulls three times, with at least 24 h between sessions.

To automatically place landmarks which coincide with the manual landmarks, a leave-one-out approach was used. One skull was determined to be the target skull, while the remaining 30 were the training dataset. Manually placed landmarks (averages per observer over the 3 landmarking rounds) were transferred to the masked skulls by translating them into barycentric coordinates. Their location on the masked skull was calculated via a weighted sum (Barycentric coordinates) of the three closest quasi-landmarks. These were averaged over the training set and then translated back to cartesian coordinates on the target skull. As such, the relative position of the manual landmark to the quasi-landmarks on the target skull was transferred to the training skulls and compared to manual placements on these skulls. This was repeated 31 times for each skull as the target, and then averaged. Due to the process of averaging, the resulting landmark on the target skull was not always on the surface. To circumvent this issue, the landmark was projected to the closest point on the surface of the target skull. This placement was repeated using each observers’ manual landmarks individually, and an average of all observers’ landmarks.

**Figure 2:**
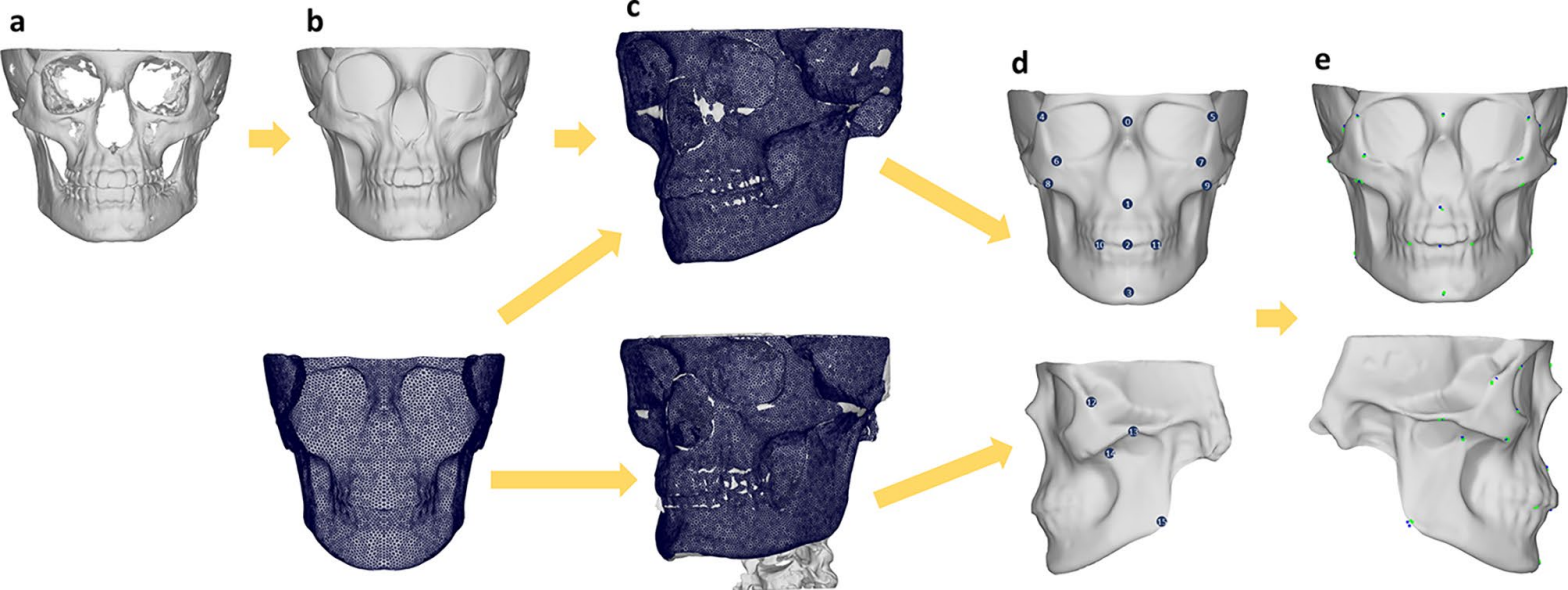
Overview of pre-assessment steps. (**a**) Skull mesh is exported from 3D slicer, (**b**) skull mesh is shrink wrapped in Blender, (**c**) craniofacial bone mask is applied to all skulls using MeshMonk, (**d**) 20 landmarks are placed manually by 3 observers, (**e**) automatic landmarks are determined.

An overview of the pre-assessment process can be found in Fig. 2.

### Reliability assessment

Intra-observer reliability of manual landmark placement was calculated as the Root Mean Square (RMS) distance (root square of the mean of squared Euclidean distances) between the three rounds of landmarking for each observer, the centroid size of the landmark configuration, as well as the standard deviation. Inter-observer reliability was calculated between the three observers over the average of their three landmarking rounds.

While automatic landmarking is consistent within itself, the MeshMonk toolbox does require the placement of initial landmarks for registration. As these are placed manually, variation can be present. Therefore, reliability of the registration, performed by observer 1 only, was assessed by calculating the RMS distance of the resulting masked quasi-landmarks to the centroid (mean point over all landmarks) over all three iterations performed. A smaller RMS shows less variation between quasi-landmark placement. The performance and reliability of the automatic landmarking was then compared with placement derived from the three observers’ manual landmarks.

To analyze if the automatic and manual landmarks were more or less variable, descriptive statistics as well as an ANOVA on centroid size with Observer, Skull, and nested Observer/Iteration was performed. MANOVA were performed on the generalized Procrustes analysis (GPA) aligned landmarks for both the manual and automatic landmarks with Skull and Observer as factors (as well as nested Observer/Iteration for the manual landmarks) to see which of these explained variation within the landmarks. Intraclass correlation coefficients (ICC) were calculated for intra and inter-observer centroid sizes (two-way consistency (inter-) and agreement (intra-).

All statistical analyses were performed either in Matlab, or in R using the packages Geomorph^39^, irr^40^, and SimplyAgree^41^. Plots were created using ggplot2^42^.

### Accuracy assessment

To calculate the accuracy of the automatic landmarks in relation to the ‘gold standard’ manual landmarks, multiple approaches were used. Initially, the Euclidean distance between the average manual and automatic landmarks were calculated to provide basic information as to which landmarks show the highest accuracy. Bland–Altman plots were used to visualize the agreement between centroid sizes. ICC statistics were used to compare landmark indications using both xyz coordinates and centroid sizes (two-way agreement). To determine if the method explained variation between the landmarks, an ANOVA was performed on centroid sizes with skull, observer, and method as predictors.

### Landmark assessment

Due to the shrink-wrapping process, gaps such as eye sockets are filled in and defined by quasi-landmarks. To calculate which of the landmarks are the best and most stable to define points on the physical skull, we calculated the distance between the MeshMonk skull and the original skull (before wrapping) along the normal vectors. Any landmarks with distances more than 10 mm in more than half of the skulls were removed. The remaining average skull was symmetrized so that the same landmarks were kept on either side. We further assessed to which extent the shrink-wrap/decimation/masking process modified the topology of the skulls by calculating the average distance along the vector normal between the original skulls and the masks.

### Image application

The craniofacial bone mask, instructions for CBCT export and shrink-wrapping, script for producing quality control images, as well as the IDs for the vertices that do not define true points on the skulls can be found on our website at https://walshlab.indianapolis.iu.edu/pages/craniofacial.html.

We also provide visualization of a basic proof of application as supplementary material where our 31 masked skulls were used to analyze sexual dimorphism and perform a Principal Component Analysis (PCA) on the solid or ‘true’ vertices (n = 6707) to show the variation attributed to sex and Principal Component (PC) 1. Each analysis was prefaced with a Partial Least Squares Regression (PLSR) to remove the effects of age, height, weight, ancestry, and sex (sex only to analyze PC1).

To illustrate that this method can also be applied to CT images, which often show a lower quality scan with more artefacts, we downloaded and processed an already cleaned CT image from the MUG500+ dataset^43^. We also evaluated the feasibility of implementing this method in a clinical setting; for this we artificially manipulated one of our skulls to display a slight deformation and removed pieces of the skull. This scan was also processed through our masking pipeline for analysis.

## Results

### Reliability

Due to the fact that our “gold standard” landmarks were placed manually, a large emphasis must be made on the intra- and inter-observer error. Table 1 shows the average RMS distance over the 20 landmarks for each observer, inter-observer, and for the automated landmarking (per landmark average, see Supplementary Table S1). RMS distances were calculated over the three MeshMonk iterations (Supplementary Fig. S3) which show that the outer rim, especially over corners (mandibular angle, nasal spine), display the most variation in the automatic landmarking. However, the error at the gonion for automatic landmarking (0.33 mm) is lower than that of manual landmarking (0.65 mm), and smaller than the lowest error (Incisors = 0.41 mm) for the trained observer 1. For automated landmarking, the majority of the central face has an error of under 0.1 mm with an average of 0.12 mm over all landmarks. The largest manual error was seen in the Zygomaxillare (2.22 mm) for both inter- and intra-observer errors. Observer 1 showed consistently smaller landmarking errors than the two other observers (Supplementary Fig. S4), resulting in a significantly lower overall RMS error (0.66 mm) in comparison to 0.94 mm and 1.11 mm, respectively. Intra-observer ICC was O1 = 0.998 (95% CI: 0.997 < ICC < 0.999), O2 = 0.988 (95% CI: 0.977 < ICC < 0.994) and O3 = 0.994 (95% CI: 0.987 < ICC < 0.997). Inter-observer ICC was 0.998 (95% CI: 0.997 < ICC < 0.999) showing high concordance in landmarking for each observer reinforcing experience as a variable is the prime factor in their choice of landmark placement and not variation during repeated measures.

**Table 1:** RMS distances (in mm) of repeated landmarking averaged over the 20 landmarks. Automatic landmarking is the average over the three rounds of MeshMonk.

	Mean	Std	Min	Max
Automated	0.119	0.086	0.052	0.347
Inter-Observer	1.442	0.462	0.738	2.393
Intra-Observer 1	0.662	0.180	0.410	1.106
Intra-Observer 2	0.943	0.267	0.627	1.494
Intra-Observer 3	1.109	0.372	0.597	2.056

An ANOVA over the centroid sizes with observer, skull and nested observer/iteration as factors showed that all factors contributed significantly to variation in centroid size (Supplementary Table S2). A MANOVA was performed on the GPA aligned manual landmarks with observer, skull, and nested observer/iteration as predictors (Supplementary Table S3). The skull itself contributed the largest amount of variation (R^2^ = 94%) while the other predictors did not contribute.

By using the observer’s manual landmarks as the “gold standard’ to compare to the automatic landmarks obtained by the pipeline, we could calculate inter-observer-directed error for the automatic landmarks. The mean standard deviation was smaller for automatic landmarks (0.77 mm) than for manual landmarks (0.902 mm). 80% of variation within the automatic landmarks was explained by individual variation, while 19% was explained by observer differences (Supplementary Table S3).

### Accuracy

#### Euclidean distance comparison

As a first measure of accuracy for the automatic landmark placement via MeshMonk, the Euclidean distance between the manual and automatic landmarks were calculated (Table 2). The average distance over all landmarks was 1.5 mm, with a range from 0.1 mm (Intercanine) to 7.2 mm (Marginal Tubercle). The variation shown on the principal axes in Supplementary Fig. S5 illustrates that often the first axis follows the contour of the skull. Landmarks with clear definition points in all axes show smaller errors while those that have a sliding placement along a contour generate larger errors. ICC for each axis is 0.998 or above showing high agreement.

**Table 2:** Descriptive statistics for the Euclidean distances between the average manual and average automatic landmarks in mm.

Landmark	Mean	Std	Min	Max
Nasion	1.079	0.677	0.223	3.301
Subspinale	1.542	0.925	0.204	4.647
Incision	0.855	0.620	0.160	3.420
Pogonion	0.844	0.551	0.146	3.491
Right Frontomalare Orbital	1.377	0.820	0.149	4.101
Left Frontomalare Orbital	1.155	0.756	0.116	3.671
Right Orbitale	1.346	0.798	0.228	3.657
Left Orbitale	1.543	0.852	0.110	4.426
Right Zygomaxillare	1.824	1.288	0.119	6.864
Left Zygomaxillare	1.811	1.345	0.103	7.021
Right Intercanine	1.083	0.724	0.100	4.573
Left Intercanine	1.068	0.754	0.110	3.892
Right Marginal Tubercle	1.820	1.220	0.285	7.246
Right Zygion	1.422	0.933	0.141	5.032
Right Koronion	2.131	1.208	0.365	6.210
Right Gonion	2.132	0.929	0.302	4.077
Left Marginal Tubercle	1.842	1.118	0.253	5.285
Left Zygion	1.485	0.939	0.128	4.175
Left Koronion	2.163	1.274	0.196	6.617
Left Gonion	1.938	1.017	0.157	4.560

#### Centroid size comparison

**Figure 3:**
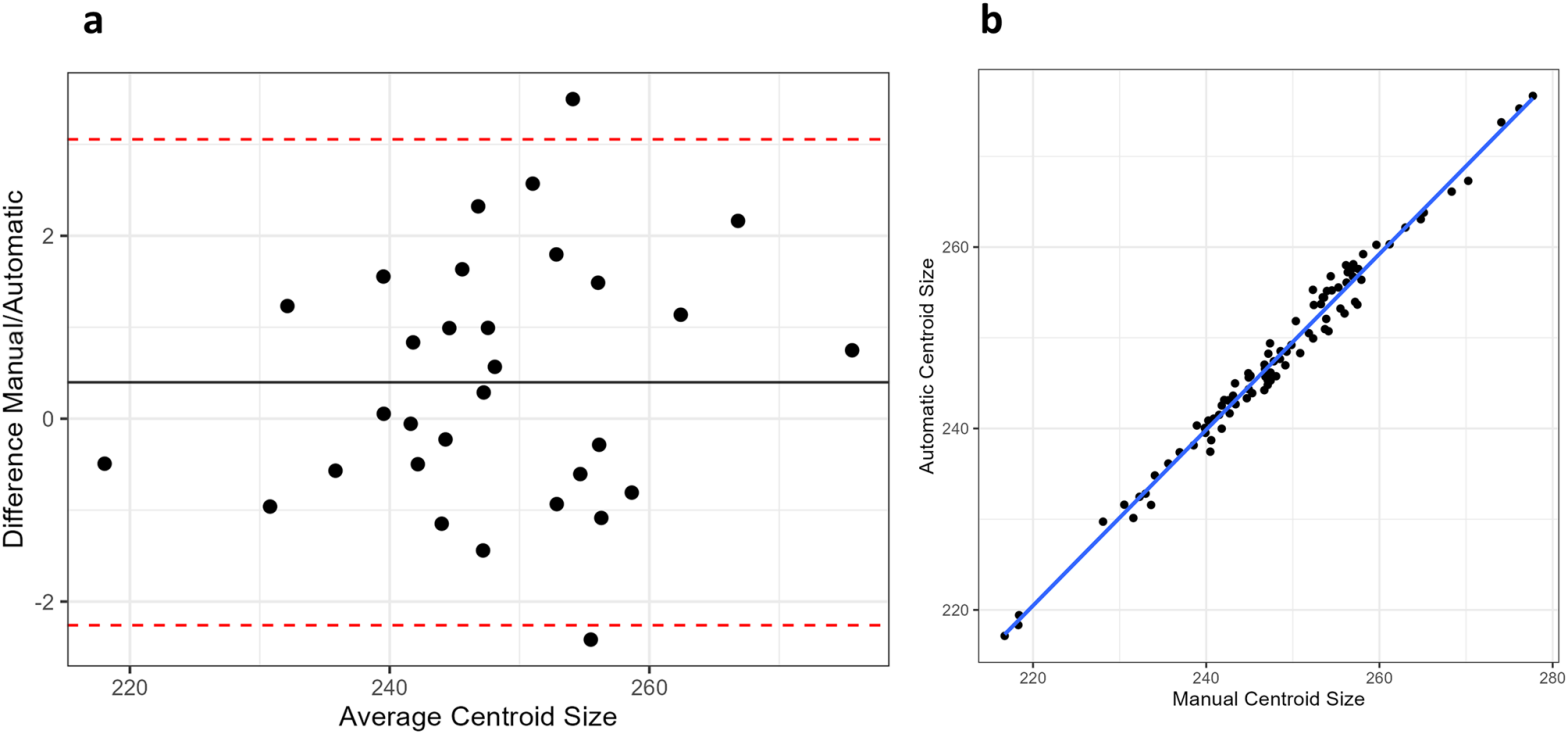
(**a**) Bland–Altman showing agreement of centroid size between manual and automatic landmark configurations. (**b**) Comparison of manual and automatic centroid sizes.

A Bland–Altman plot shows a mean difference in centroid size of 0.4 mm between the two methods, in addition to high concordance between centroid sizes over all the CBCT skulls (Fig. 3). ICC calculated from centroid sizes was 0.99 (95% CI: 0.986 < ICC < 0.994) showing negligible differences in the landmarking method. An ANOVA on centroid size with skull and method as factors showed that the method itself was not significant (p = 0.06), while skull and observer were highly significant (p < 0.001) (Table 3).

**Table 3:** ANOVA on centroid size of manual and automatic landmark configurations. Skull and method were inputted as factors.

	Df	Sum Sq	Mean Sq	F value	Pr (> F)
Skull	30	22,825.48	760.849	958.120	< 0.001
Observer	2	241.90	120.951	152.311	< 0.001
Method	1	2.84	2.844	3.581	0.062
Skull × Observer	60	20.13	0.336	0.423	1.000
Residuals	92	73.06	0.794		

### Established landmarks and successful image application

**Figure 4:**
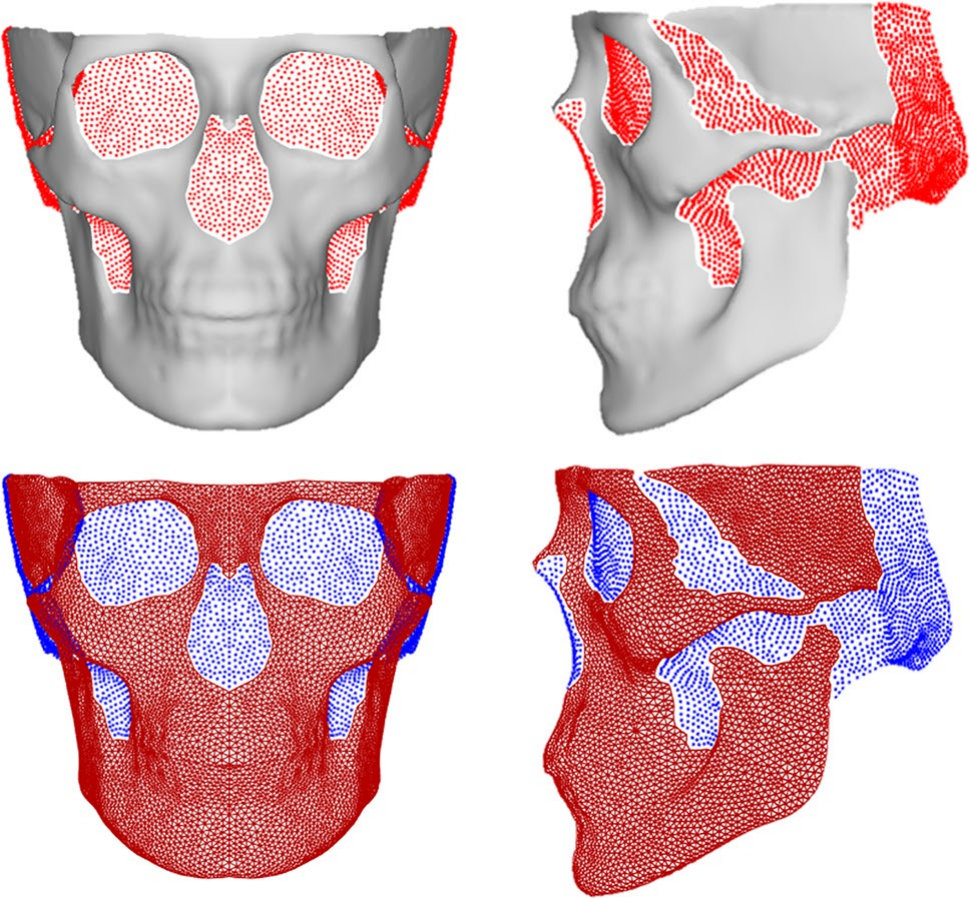
Visual representation of the “true” stable landmarks and the “gap” landmarks. Vertex IDs for these landmarks can be found in the supplementary file.

Of the 9999 original quasi-landmarks, 6707 were defined as solid or “true” landmarks pertaining to locations on the physical skull. Those landmarks that fill gaps were flagged and can visually be seen to locate on (1) gaps such as the eye sockets and nose, (2) areas near the back of the skull that are not well represented in CBCT images (Fig. 4). An assessment of the average distance between the original skull and the mask showed a 0.3 mm ± 0.3 mm difference in topology (Supplementary Fig. S6). Larger distances were found around the borders of the mesh, and between the teeth. A basic analysis of sexual dimorphism and PCA was performed on all 6707 landmarks and can be found in the Supplementary Material (GIF 1 (Sex) and 2 (PC1)) as well as our website https://walshlab.indianapolis.iu.edu/pages/craniofacial.html. We also provide information on which landmarks are “true” so that our mask (to note; user-created masks must be calculated separately) can be easily cropped to the relevant area. The CT image taken from the MUG500+ dataset^43^ was also successfully masked, as was a skull with missing areas and deformations (Supplementary Fig. S7) to show proof of application on other types of facial scans as well as structures outside normal-range.

## Discussion

While our understanding of human facial morphology and its variation has made notable progress, particularly with regards to soft tissue variation, our knowledge of the underlying hard tissue structures has significantly trailed. This disparity can be primarily attributed to the paucity of extensive skull datasets and the limited development of advanced 3D morphometric methods for skull shape analysis. To overcome this, the utilization of CBCT imagery, typically taken by dentists provides a more accessible solution whilst offering superior resolution in the form of skull mesh reconstructions. However, the analysis of CBCT-derived skull data is not devoid of inherent challenges; they can exhibit only partial cranial representations and are characterized by the absence of posterior and superior cranial segments, alongside potential holes in the mesh structure. A consequence of this is that 3D masks must be designed and adjusted to accommodate these challenging structural limitations. In addition, the meshes can be irregular, and prone to artifacts stemming from minor movements, dental interventions, and the utilization of head supports during imaging procedures. Lastly, CBCT skull meshes incorporate all hard tissue, including structures within the skull, making these meshes complex and difficult to mask. CT images show similar issues: while the mesh is often of the complete skull, artefacts are more extreme, and the surface texture is more irregular.

With the aim of enhancing the manageability of more difficult hard tissue scans, we designed and provide a craniofacial template bone mask that not only reduces the complexity of skull scans by targeting a specific area of interest but also leverages a previously proven 3D phenotyping methodology^28^. Through the process of shrink-wrapping and subsequent reduction of the skull meshes derived from Cone Beam Computed Tomography (CBCT) and Computed Tomography (CT) imagery, we achieve a substantial reduction in polygon count, facilitating more manageable data handling, particularly on less robust computational platforms. The innovative approach of wrapping the skulls also addresses some of the scan complications described above whilst permitting MeshMonk, an open-source tool, to mask the skull. While the use of MeshMonk on a full skull has been published^37^, this application could not be tested on our CBCT or CT scans as the template is not freely available. The authors also note that the mask consisted of ~ 177,000 quasi-landmarks, necessitating significant computational resources, and the use of the full skull captured using the same imaging modality.

In addition, we aimed to evaluate the reliability and accuracy of our freely available bone mask in comparison to manual landmark placement, as observer errors are often large, especially when lacking experience. Firstly, we considered the intra- and inter-observer errors associated with manual landmarking of the skull to give a baseline. 20 landmarks were selected that were easily identifiable on the skull. Three different levels of experience were chosen to highlight the need for training with manual landmark placement, something that is less important when using the automatic landmarking tools designed and presented here. The manual landmark values are similar to those reported in other studies^28,31,37^, albeit slightly higher than findings from a study employing specialized landmarking software at some landmarks^37^. As expected, the inter-observer error was higher (1.44 mm). After processing through the automatic landmarking method, this error was compared to the inter-observer error, revealing a more than 6× smaller (0.12 mm) result. Hence, the use of the automated landmarking mask can significantly reduce inherent manual placement error across the entire skull.

Errors in automatic landmark placement may be due to variations in the placement of the few manually placed initial landmarks required by MeshMonk. However, these errors predominantly manifest along the periphery of the mask and correspond to regions that may not always be entirely captured in CBCT scans. Specifically, the absence of posterior segments can result in the mask coalescing in this region. This is especially visible when calculating the “true” landmarks as it was evident these posterior points were not well represented in our cohort and thus flagged as “gaps”. Consequently, it is plausible that this issue may be more attributable to limitations inherent to the CBCT imaging technique rather than deficiencies in the masking methodology. Notably, the maximum MeshMonk error is higher than that seen for a mandible mask^31^, albeit confined to these specific regions. Consequently, the effect of initial manual landmarking error is balanced out by the MeshMonk mask registration. Moreover, MeshMonk still offers over 6000 landmarks in a fraction of the time required for the manual placement of 20 performed by observers, and at a lower error rate overall.

To explore and mitigate bias on the automation, a leave-one-out approach was used. Analysis revealed a variation in Euclidean distance between manual and automatic landmarks ranging from 0.10 to 7.24 mm at the 20 landmarks explored. Notably, landmarks exhibiting higher values were often associated with regions that were less effective during the masking process (e.g., gonion where the mask did not always align with the edge of the skull), and were prone to higher inter-observer errors, as seen in previous studies^31^. Previous research has reported comparable errors between manual and automatic landmark placements ranging from 2.19 mm^37^, 1.4 mm^31^, 2.01 mm^44^ and 1.26 mm^28^, with our study revealing a similar finding of an average of 1.5 mm. ICC values consistently demonstrated high levels of agreement (ICC > 0.9) for automation. Interestingly, observer-related variability contributed to 19% of the variation in automatic landmark placement when testing these 20 landmarks, but this factor was negligible in the case of manual placement. It makes sense that this observation supports our finding that variation introduced by different observers can have an impact on testing the automation, as manual placements are used to calculate the difference in automatic landmark placement. Notably, a considerably smaller proportion of variance remained unexplained for the automatic landmarks (0.4% vs. 6%). An ANOVA on centroid size also demonstrated that the method employed did not exert a significant influence on the variation observed in landmark configurations or shape. As such, MeshMonk in combination with our mask provides a larger number of quasi-landmarks with a high level of reliability and accuracy.

However, both the masking process and the analysis process have some limitations. The conventional use of manual landmarks as the “gold standard” has been applied and observed in previous studies^28,31,45^, yet it raises certain inherent issues. Specifically in this case as two out of the three observers lacked prior extensive training in landmarking. This introduces more variability in the automatic landmark placement. Moreover, our analysis focused on a limited set of 20 landmarks, therefore the accuracy of the remaining quasi-landmarks was not systematically assessed. However, given the low error associated with automatic landmarking, it is reasonable to assume that these additional landmarks exhibit a comparable level of accuracy, which remain significantly lower than manual landmarking errors. Another limiting factor was the quality of the CBCT images. Despite our efforts to select images with minimal artifacts, many still exhibited minor missing portions and common CBCT imaging artifacts. Additionally, there was considerable variability in the extent of image coverage, particularly in the posterior regions of the scans due to the CBCT machines’ limited FOV. Thus, it was expected that our results showed higher errors than commonly seen when landmarking complete 3D skull scans or physical skulls. However, these types of scans represent the reality of present data sets. It also underscores the viability of the shrink-wrapped craniofacial bone mask as an effective method that more easily facilitates the comparison of the human skulls geometric shape, even with deformations and missing areas, when derived from CBCT and CT imagery.

CBCT scans typically comprise high polygon meshes abundant in detail. Through shrink-wrapping and decimation processes, we decrease their resolution and potentially alter their topology. Although our analysis indicates minimal error across most regions of the skull, enhancing resolution or bypassing decimation steps could further ameliorate this, yet at the cost of significantly increased time and computational resources. Furthermore, reduced resolution and shrink-wrapping complicate the identification of anatomical landmarks on the skull. Nevertheless, corresponding positions on the mask can be determined for these landmarks, removing the need for manual landmark assessment. Although this craniofacial bone mask may only cover a portion of the cranium, it facilitates applicability to CBCTs acquired from devices with limited focal views. In the future we plan to extend this work to a full cranial mask and utilize the development of superior CBCT scanners^46,47^ and deep learning for DICOM segmentation^48^. Although the mask does provide over 6000 quasi-landmarks, it is limited by the less-accurate masking of the gonial area. This, in particular, is important in dental work, therefore optimization of the Mesh Monk settings and advancements in non-rigid registration could help mitigate this issue.

Many research groups working on geometric morphometrics and the genetics behind skull and face shape may lack formal anthropological/anatomical training or landmarking experience, leading to elevated manual landmarking errors akin to those observed with observer 3. By providing a method for stable automatic landmarking, this error is minimized. It also reduces time investment, enhances objectivity, and has the capacity to analyze a greater number of landmarks. Additionally, the deliberate focus on the external aspects of the skull and the creation of a single-plane craniofacial bone mask not only reduces computational resource requirements but also standardizes subsequent analyses. We have also supplied a comprehensive workflow for the shrink-wrapping procedure, quality control scripts, and the vertex IDs for the stable landmarks, rendering this method easily adaptable in research laboratories without the need for specialized training. The adoption of a standardized mask further facilitates the efficient comparison of data across various studies, including the medical and dental fields. It facilitates investigations into phenomics and explorations of the genetic and anatomical underpinnings of facial shape. Given that our mesh is open-source, information can be readily exchanged among groups utilizing the same mask, substantially augmenting sample sizes and/or standardizing thousands of landmark placements across patient samples to aid in diagnostics. To visualize these potential applications, we provide a PCA and sexual dimorphism analysis that could easily show morphological variation even within our modest sample set. Overall, we feel the greatest benefit lies in the potential for comparability between research groups and the establishment of extensive databases capable of utilizing the same mask on both CBCT and CT scans for future craniofacial applications.

## Conclusion

Within this study we designed and provide a freely available 3D craniofacial template bone mask for the dense 3D phenotyping of skull meshes exported from CBCT/CT scans in addition to a tutorial outlining the procedure for preparing these images for masking. The provided template can be used within the MeshMonk framework, facilitating the generation of high-density cranial landmarks for subsequent analyses with minimal manual intervention and in a notably efficient manner. Our methodology has demonstrated a high level of accuracy, with substantially reduced errors when compared to manual landmark placement. This standardized approach not only enhances reliability and precision but also minimizes the potential for errors in landmark identification and placement on hard tissue structures of the human face. Ultimately, we envision that our work will pave the way for genetic association studies pertaining to cranial shape and high-resolution investigations into the genetic determinants influencing craniofacial bone morphology.

### Supplementary Information

Supplementary Information 1.

Supplementary Information 2.

Supplementary Information 3.

## Data Availability

The craniofacial template bone mask that is the basis of this work can be downloaded from our website; https://walshlab.indianapolis.iu.edu/pages/craniofacial.html. The MeshMonk (v.0.0.6) spatially dense facial-mapping software is provided by KU Leuven and is free to use for academic purposes (https://gitlab.kuleuven.be/mirc/MeshMonk). All code used for analyses was modified from a previous publication (10.1038/s41588-020-00741-7). Further underlying code is available as part of the Matlab software (https://www.mathworks.com) as well as 3D Slicer (https://www.slicer.org/), Blender (https://www.blender.org/), Meshmixer (https://meshmixer.com/) and MeVisLab (https://www.mevislab.de/).
