# Supplementary figures and images for: A novel approach to craniofacial analysis using automated 3D landmarking of the skull
Source: Sci Rep. 2024 May 29;14:12381. doi: 10.1038/s41598-024-63137-1 (PMC11137148; doi:10.1038/s41598-024-63137-1)

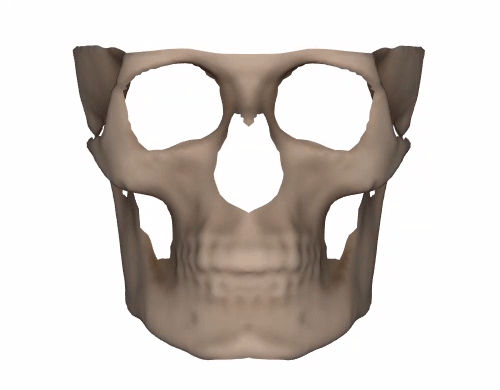

Supplement: Supplementary file 1 — Supplementary Information 1. [file 41598_2024_63137_MOESM1_ESM.gif]

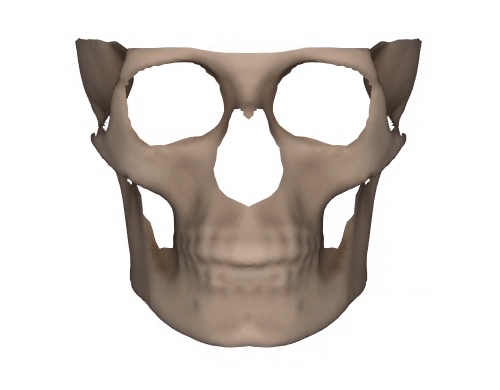

Supplement: Supplementary file 2 — Supplementary Information 2. [file 41598_2024_63137_MOESM2_ESM.gif]
