# Supplementary material for: A novel approach to craniofacial analysis using automated 3D landmarking of the skull
Source: Sci Rep. 2024 May 29;14:12381. doi: 10.1038/s41598-024-63137-1 (PMC11137148; doi:10.1038/s41598-024-63137-1)

**SUPPLEMENTARY INFORMATION**

**TITLE**

A Novel Approach to Craniofacial Analysis using Automated 3D Landmarking of the Skull

**AUTHORS**

Franziska Wilke^1^, Harold Matthews^2,3,8^, Noah Herrick^1^, Nichole Dopkins^1^, Peter Claes^2,3,8,9^, Susan Walsh^1*^

^1^ Department of Biology, Indiana University Indianapolis, Indianapolis, USA.

^2^ Department of Human Genetics, KU Leuven, Leuven, Belgium.

^3^ Murdoch Children’s Research Institute, Melbourne, Victoria, Australia.

^4^ Department of Human Genetics, University of Pittsburgh, Pittsburgh, PA, USA.

^5^ Center for Craniofacial and Dental Genetics, Department of Oral and Craniofacial Sciences, University of Pittsburgh, Pittsburgh, PA, USA.

^6^ Department of Anthropology, University of Pittsburgh, Pittsburgh, PA, USA.

^7^ Department of Anthropology, The Pennsylvania State University, University Park, PA, USA.

^8^ Medical Imaging Research Center, University Hospitals Leuven, Leuven, Belgium.

^9^ Department of Electrical Engineering, ESAT/PSI, KU Leuven, Leuven, Belgium.

*Corresponding Author:

Susan Walsh

Department of Biology, Indiana University Indianapolis, 723 W Michigan St, Indianapolis, IN 46202, USA.

walshsus@iu.edu


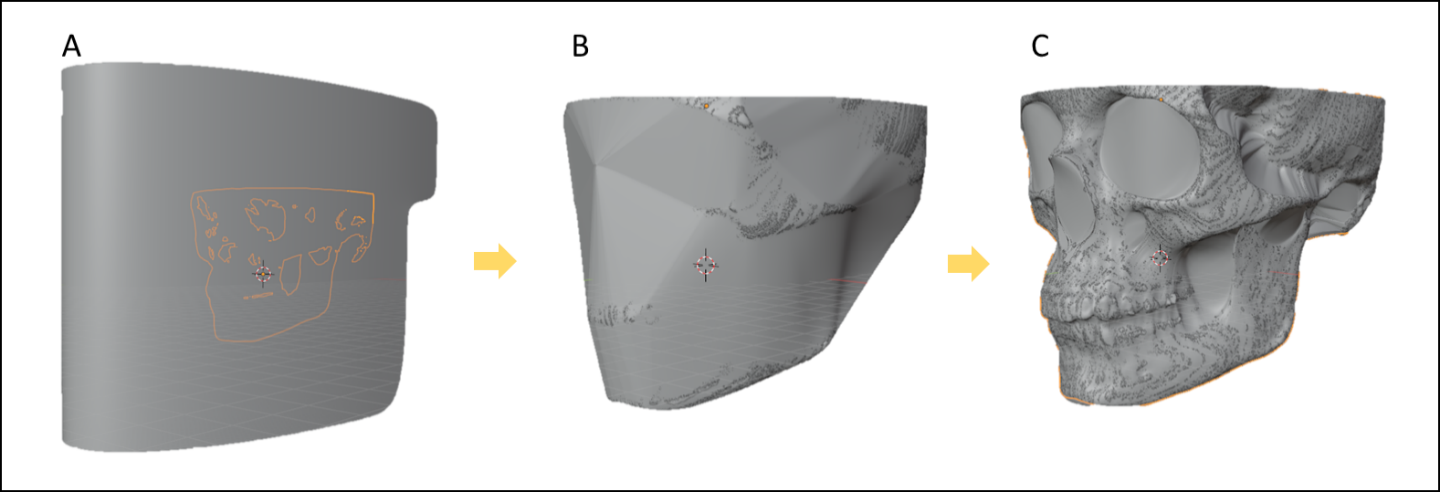
**Supplementary Figure S1:** Shrink-wrapping in Blender. A: a deformed half cylinder is placed around the skull, B: using Blender’s shrink-wrapping tool the cylinder is wrapped to the skull, C: This step is repeated 6x while applying the subdivision surface modified in between rounds.


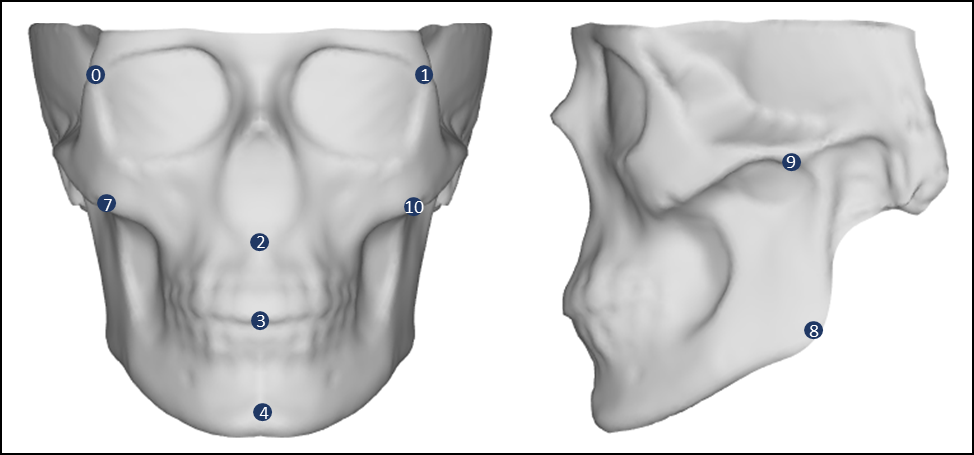
**Supplementary Figure S2:** Landmarks used for MeshMonk initialization.

| **Landmark** | **O1** | **O2** | **O3** | **IO** | **MM** |
| --- | --- | --- | --- | --- | --- |
| Nasion | 0.569 | 0.897 | 0.727 | 1.610 | 0.052 |
| Subspinale | 0.657 | 0.903 | 0.812 | 1.498 | 0.290 |
| Incision | 0.410 | 0.656 | 0.597 | 0.920 | 0.115 |
| Pogonion | 0.459 | 0.658 | 0.730 | 1.788 | 0.077 |
| Right Frontomalare Orbital | 0.483 | 0.790 | 0.734 | 1.464 | 0.092 |
| Left Frontomalare Orbital | 0.531 | 0.703 | 0.836 | 1.977 | 0.079 |
| Right Orbitale | 0.806 | 1.234 | 0.984 | 1.792 | 0.122 |
| Left Orbitale | 0.918 | 0.918 | 0.899 | 1.653 | 0.076 |
| Right Zygomaxillare | 1.106 | 1.388 | 2.056 | 2.393 | 0.122 |
| Left Zygomaxillare | 0.980 | 1.494 | 1.805 | 2.053 | 0.124 |
| Right Intercanine | 0.684 | 1.182 | 1.076 | 1.448 | 0.086 |
| Left Intercanine | 0.688 | 1.202 | 0.911 | 1.311 | 0.095 |
| Right Marginal Tubercle | 0.455 | 0.627 | 1.123 | 0.880 | 0.059 |
| Right Zygion | 0.779 | 1.080 | 1.603 | 1.948 | 0.080 |
| Right Koronion | 0.600 | 0.816 | 1.058 | 0.738 | 0.058 |
| Right Gonion | 0.577 | 0.723 | 1.071 | 0.914 | 0.322 |
| Left Marginal Tubercle | 0.511 | 0.695 | 1.193 | 0.946 | 0.065 |
| Left Zygion | 0.662 | 1.344 | 1.369 | 1.598 | 0.068 |
| Left Koronion | 0.634 | 0.812 | 1.157 | 0.850 | 0.052 |
| Left Gonion | 0.730 | 0.746 | 1.434 | 1.069 | 0.347 |
| Mean | 0.662 | 0.943 | 1.109 | 1.442 | 0.119 |
| Std | 0.180 | 0.267 | 0.372 | 0.462 | 0.086 |
| Min | 0.410 | 0.627 | 0.597 | 0.738 | 0.052 |
| Max | 1.106 | 1.494 | 2.056 | 2.393 | 0.347 |

**Supplementary Table S1:** Intra-Observer (O1-O3) and Inter-Oberver (IO) error shown as the RMS distance (in mm) to the centroid over the 3 landmarking rounds/three observers per landmark, as well as over the three MeshMonk iterations (MM).

**Supplementary Figure S3:** RMS distance (in mm) to centroid over the three MeshMonk iterations for all 9,999 quasi-landmarks.

|  | **Mean** | **95% CI Mean** | | **SD** | **Min** | **Max** |
| --- | --- | --- | --- | --- | --- | --- |
| **Automated** | 0.1190 | 0.0769 | 0.1611 | 0.0899 | 0.0519 | 0.3468 |


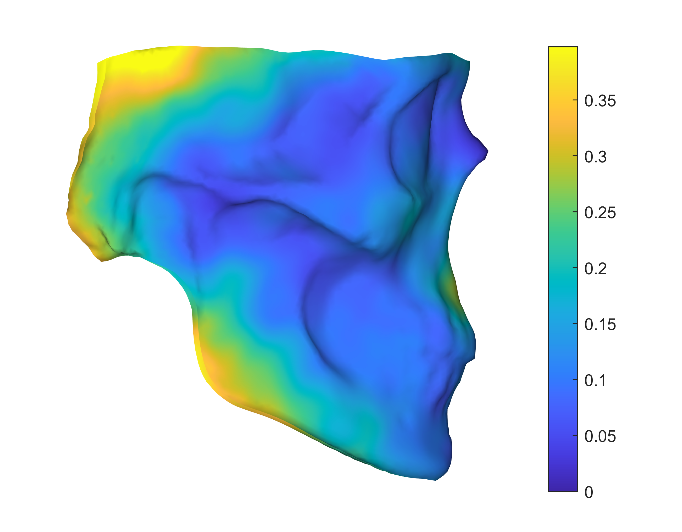

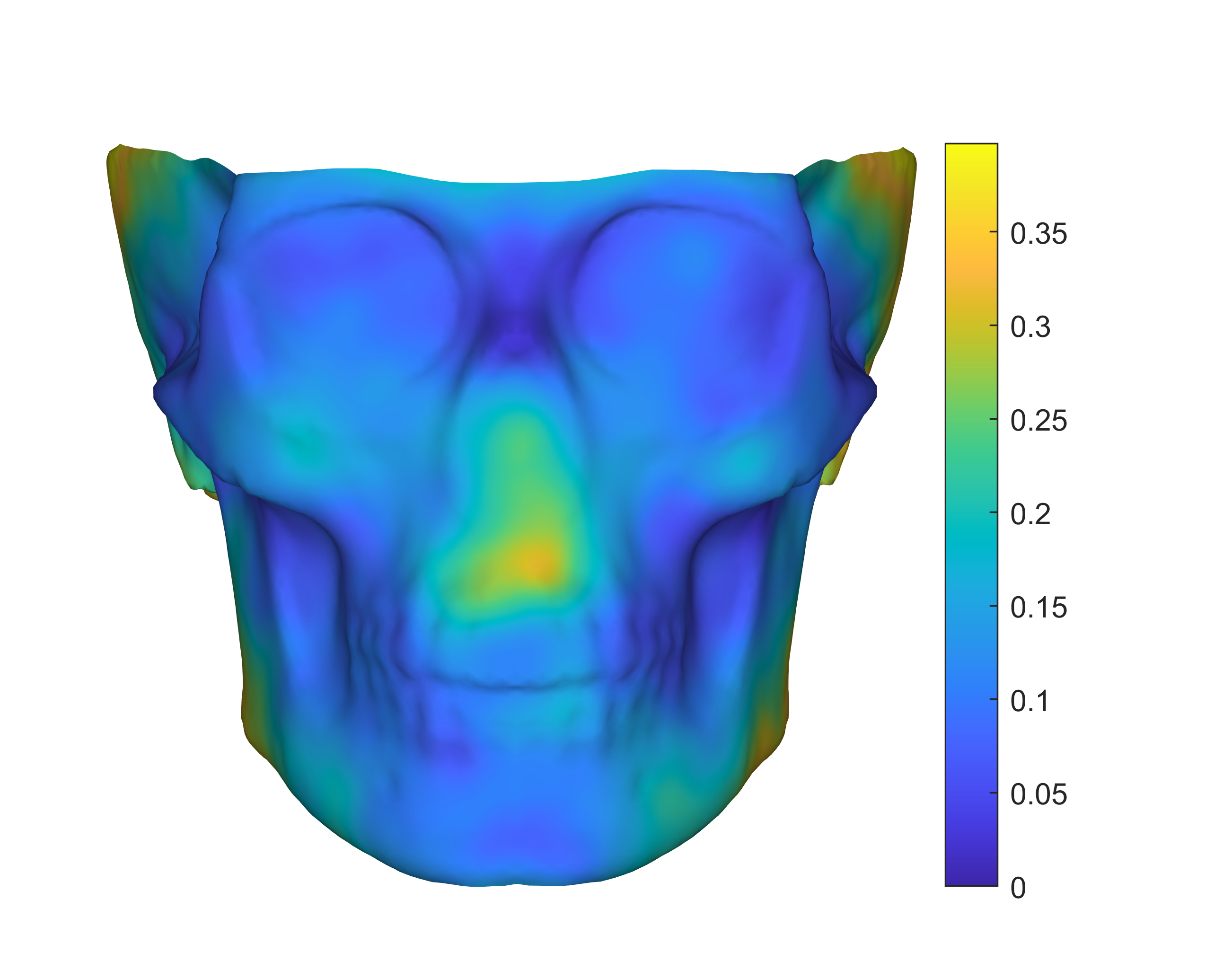

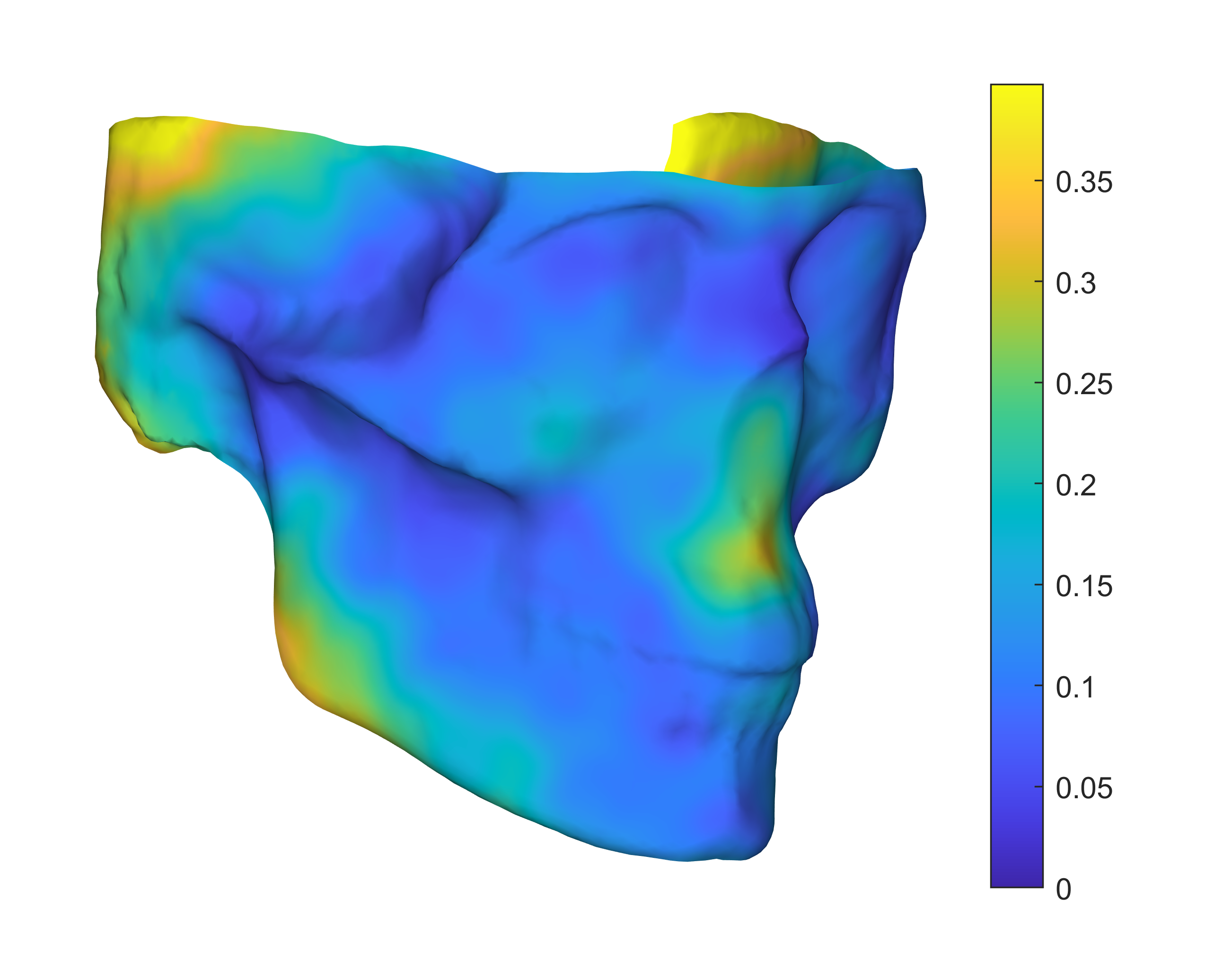


**
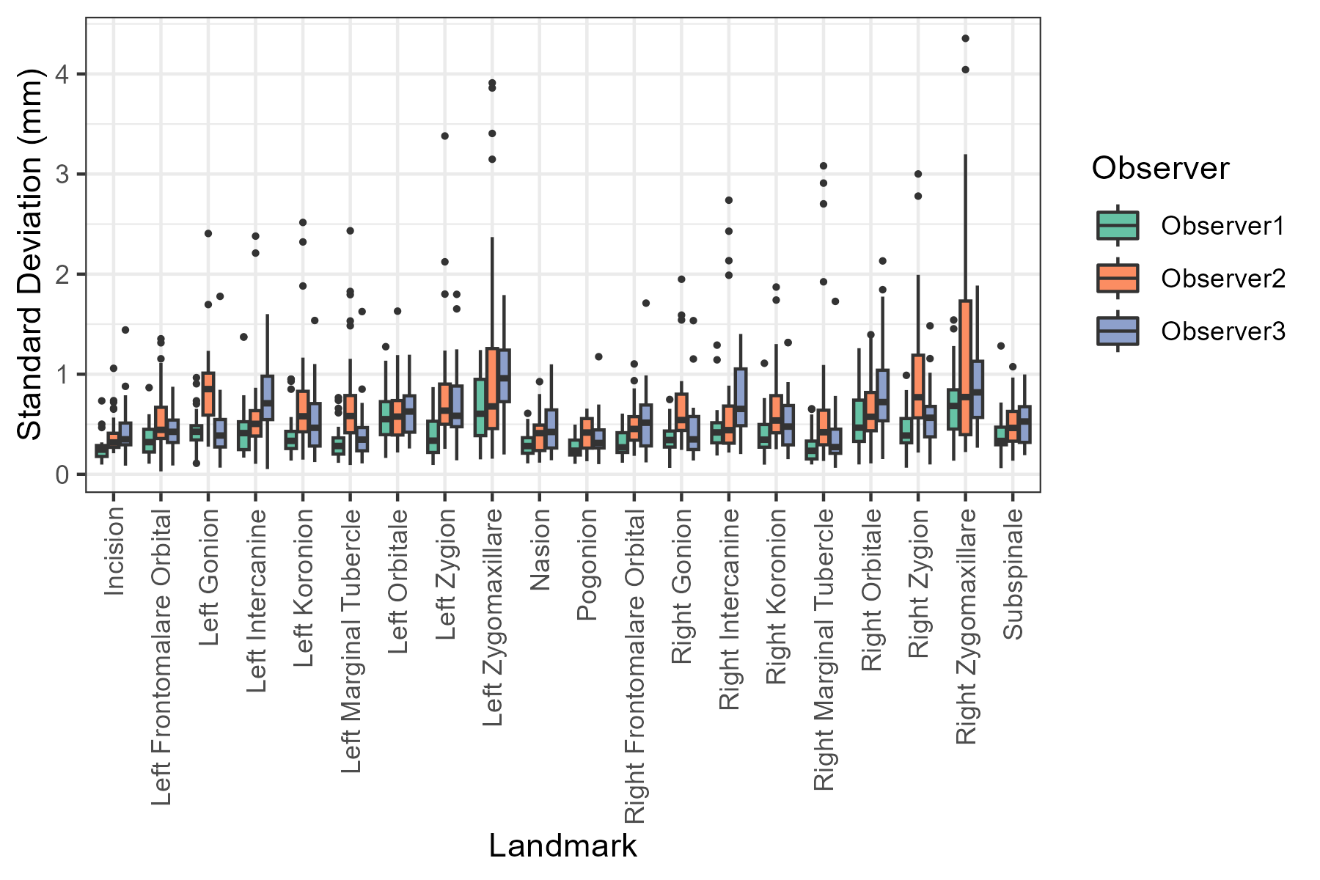
Supplementary Figure S4:** Boxplot of intra- and inter-observer errors shows as the standard deviations in mm over the three landmarking iterations/observers averaged over the three axes per landmark.


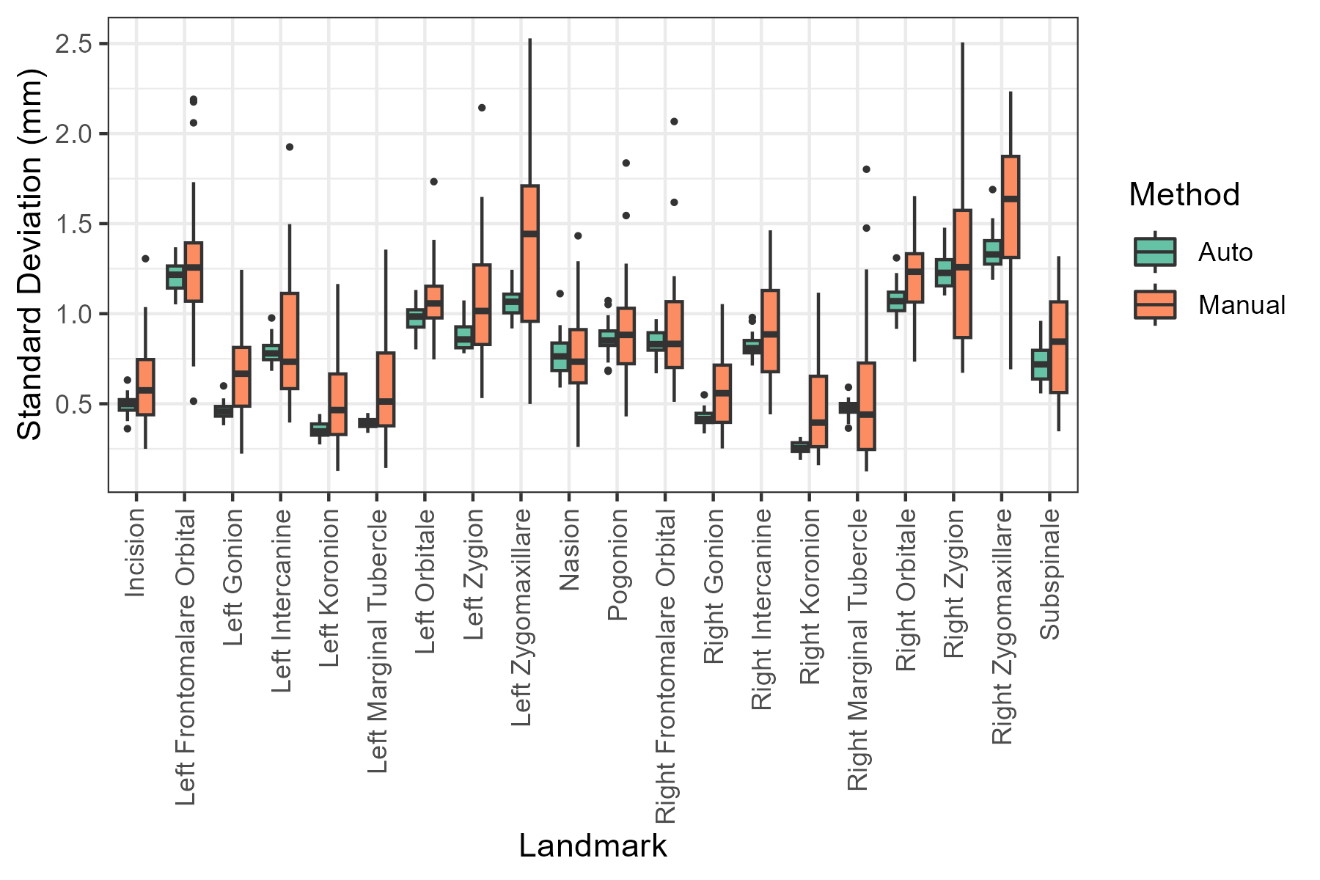


**Supplementary Table S2:** ANOVA on the centroid sizes for all manual landmarks. Skull, observer, and nested observer/iteration were inputted as factors.

|  | **Df** | **Sum Sq** | **Mean Sq** | **F value** | **Pr(>F)** |
| --- | --- | --- | --- | --- | --- |
| **Skull** | 30 | 34417.448 | 1147.248 | 1507.151 | <0.001 |
| **Observer** | 2 | 379.337 | 189.669 | 249.170 | <0.001 |
| **Skull*Observer** | 60 | 105.202 | 1.753 | 2.303 | <0.001 |
| **Observer*Iteration** | 6 | 23.672 | 3.945 | 5.183 | <0.001 |
| **Residuals** | 180 | 137.017 | 0.761 |  |  |

**Supplementary Table S3:** MANOVA on the GPA aligned manual and auto landmarks. Skull, observer, and nested observer/iteration (for manual only) were inputted as predictors.

| **MANUAL** | **Df** | **SS** | **MS** | **Rsq** | **F** | **Z** | **Pr(>F)** |
| --- | --- | --- | --- | --- | --- | --- | --- |
| **Observer** | 2 | 0.0000 | 0.0000 | 0.0000 | 0.000 | -9.801 | 1 |
| **Skull** | 30 | 0.7195 | 0.0240 | 0.9396 | 96.396 | 5.409 | 0.01 |
| **Observer*Skull** | 60 | 0.0000 | 0.0000 | 0.0000 | 0.000 | -9.950 | 1 |
| **Residuals** | 186 | 0.0463 | 0.0002 | 0.0604 |  |  |  |
| **Total** | 278 | 0.7658 |  |  |  |  |  |

| **AUTOMATIC** | **Df** | **SS** | **MS** | **Rsq** | **F** | **Z** | **Pr(>F)** |
| --- | --- | --- | --- | --- | --- | --- | --- |
| **Observer** | 2 | 0.0448 | 0.0224 | 0.1946 | 1254.732 | 7.908 | 0.01 |
| **Skull** | 30 | 0.1841 | 0.0061 | 0.8007 | 344.108 | 9.915 | 0.01 |
| **Residuals** | 60 | 0.0011 | 0.0000 | 0.0047 |  |  |  |
| **Total** | 92 | 0.2300 |  |  |  |  |  |

**Supplementary Figure S5:** Visual and tabular variation of the Euclidean distance error over the 20 manually placed landmarks between manual and automatic landmarks. Variation is shown as the three largest dimensions and in which direction this variation occured.

| **Landmark** | **Red (dim1)** | **Blue (dim2)** | **Green (dim3)** |
| --- | --- | --- | --- |
| Nasion | 1.168 | 0.422 | 0.273 |
| Subspinale | 1.629 | 0.673 | 0.343 |
| Incision | 0.813 | 0.525 | 0.418 |
| Pogonion | 0.876 | 0.426 | 0.253 |
| Right Frontomalare Orbital | 1.479 | 0.560 | 0.241 |
| Left Frontomalare Orbital | 1.215 | 0.605 | 0.239 |
| Right Orbitale | 1.311 | 0.807 | 0.266 |
| Left Orbitale | 1.614 | 0.662 | 0.231 |
| Right Zygomaxillare | 2.071 | 0.719 | 0.402 |
| Left Zygomaxillare | 2.057 | 0.844 | 0.351 |
| Right Intercanine | 1.107 | 0.606 | 0.315 |
| Left Intercanine | 1.028 | 0.730 | 0.333 |
| Right Marginal Tubercle | 2.036 | 0.661 | 0.449 |
| Right Zygion | 1.533 | 0.679 | 0.266 |
| Right Koronion | 2.128 | 1.033 | 0.625 |
| Right Gonion | 1.883 | 1.227 | 0.591 |
| Left Marginal Tubercle | 2.018 | 0.610 | 0.428 |
| Left Zygion | 1.592 | 0.689 | 0.261 |
| Left Koronion | 2.101 | 1.232 | 0.595 |
| Left Gonion | 1.816 | 1.151 | 0.393 |


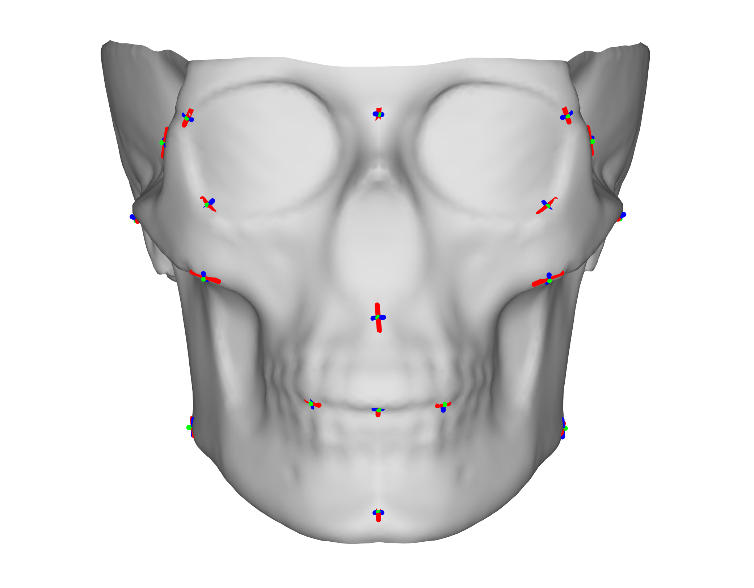

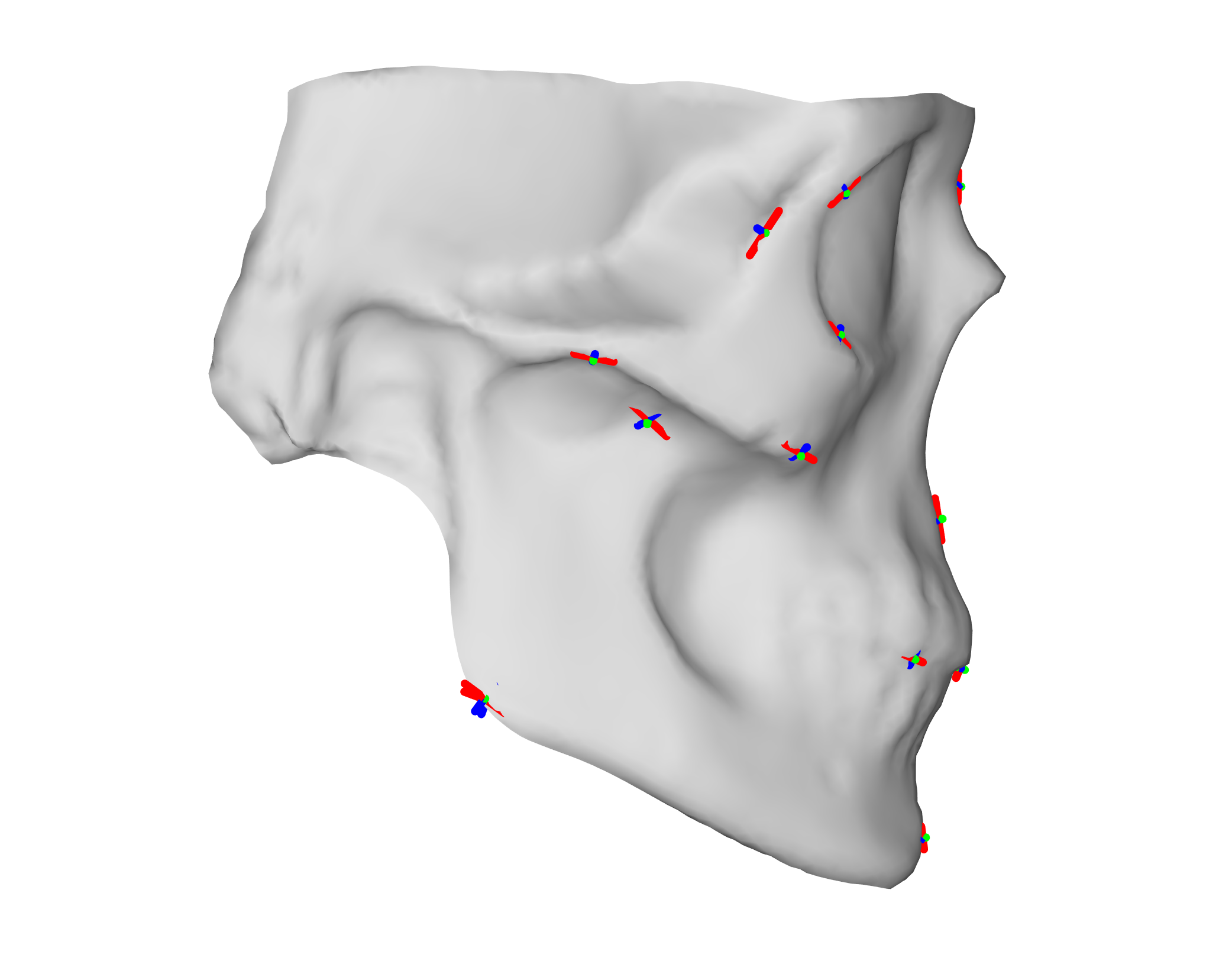

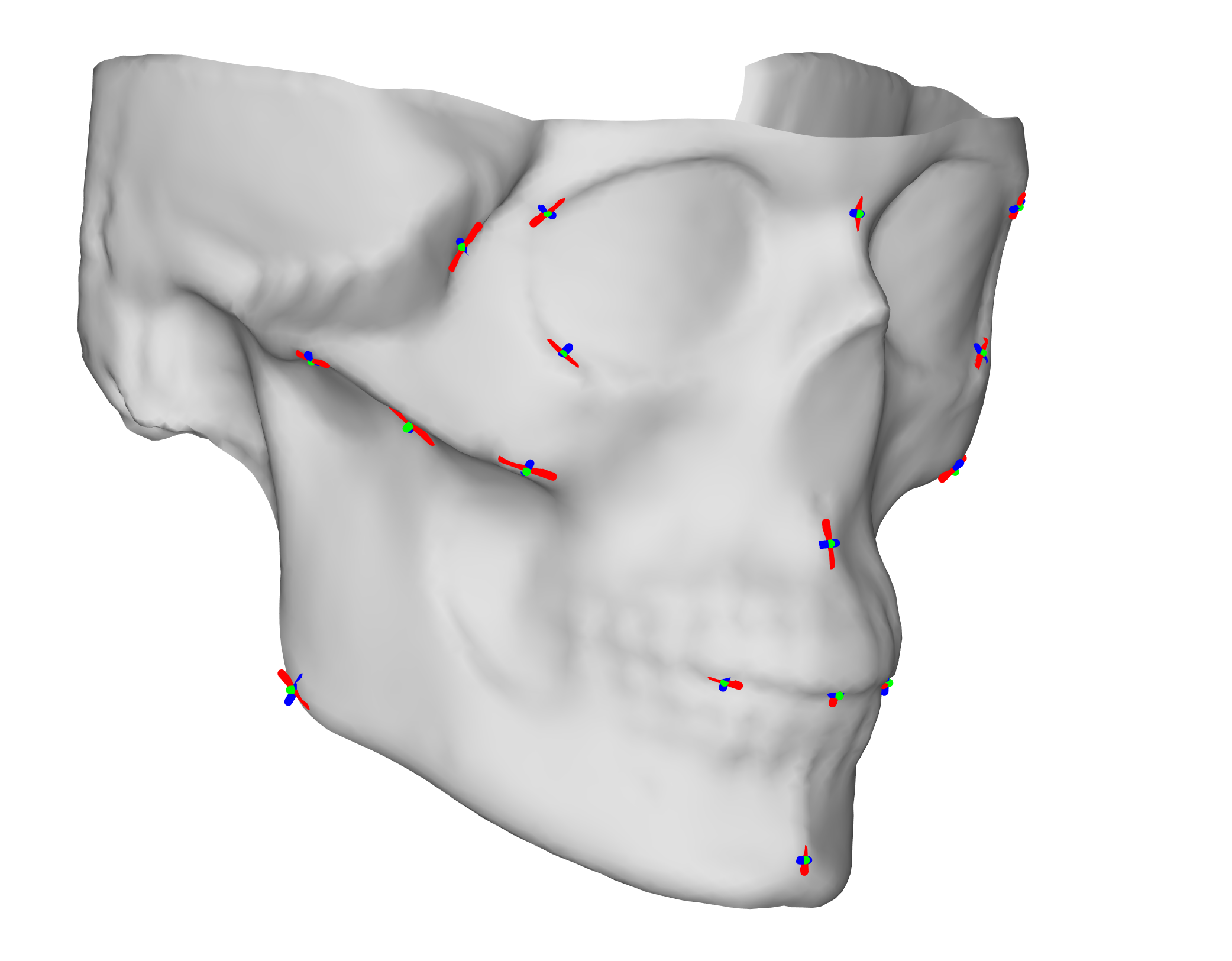


**Supplementary Figure S6:** Heatmap to show the A: average distance range (in mm) and B: standard deviation range (in mm) between the original skull exported from 3D Slicer and the resulting masked skull.


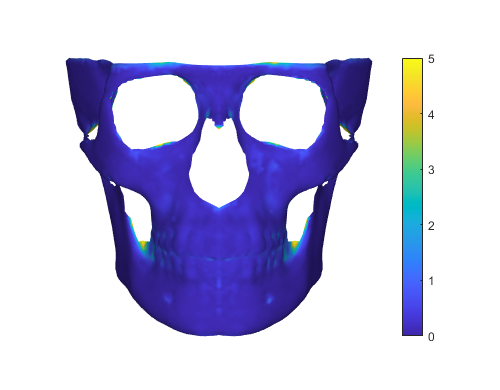

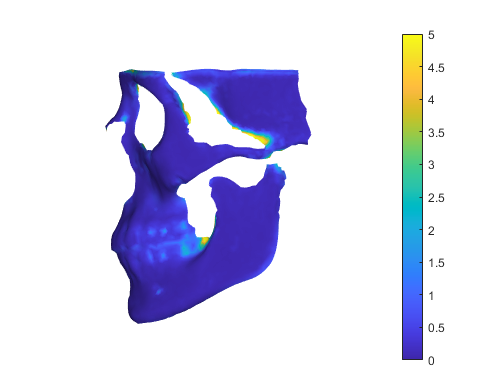

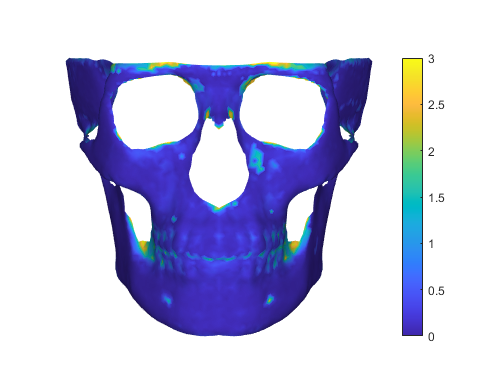

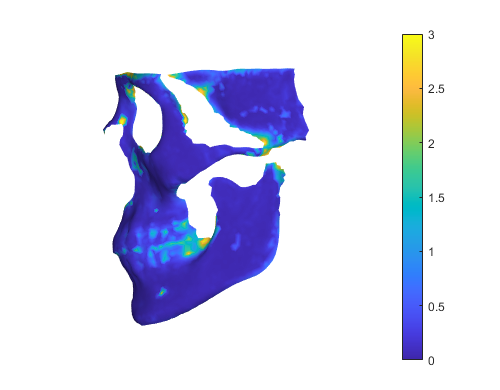


A

B

**Supplementary Figure S7:** Visualization of the masking process on a CT scan, and on a deformed and incomplete skull. A: cleaned CT scan from MUG500+, B: After shrinkwrapping, C: After decimation/remeshing, D: After masking.


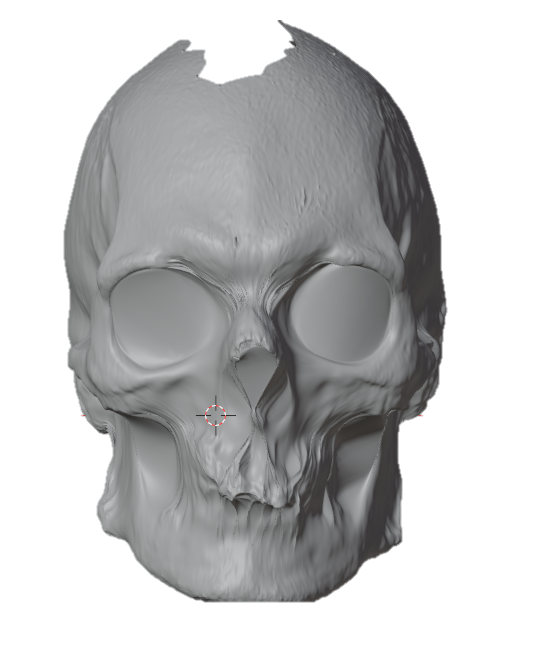

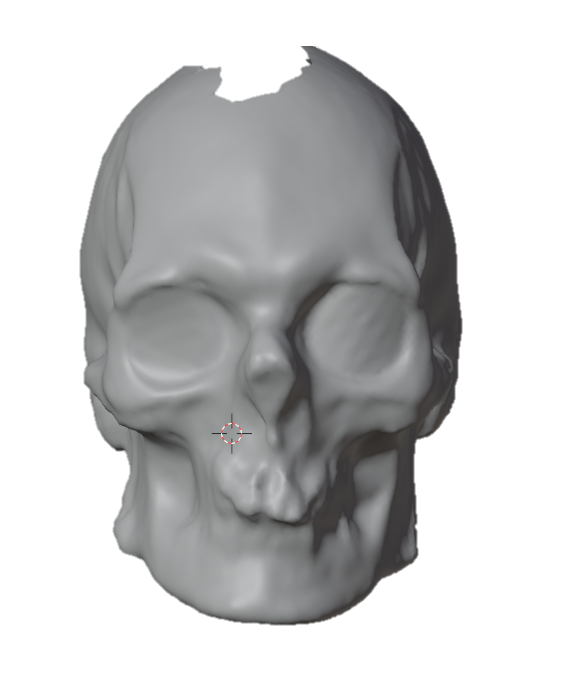

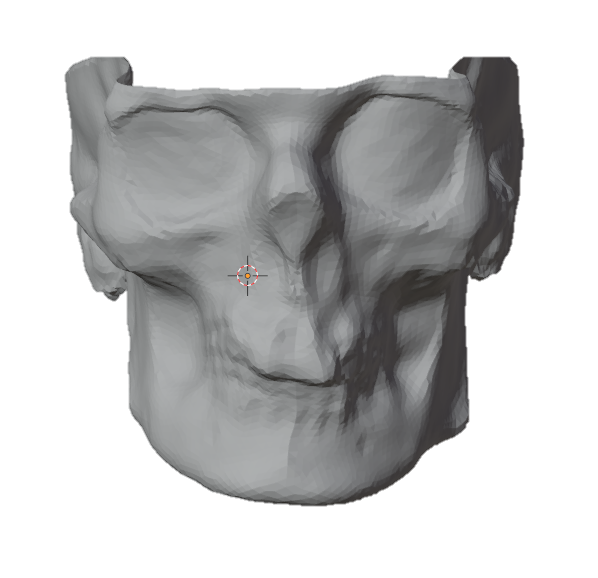

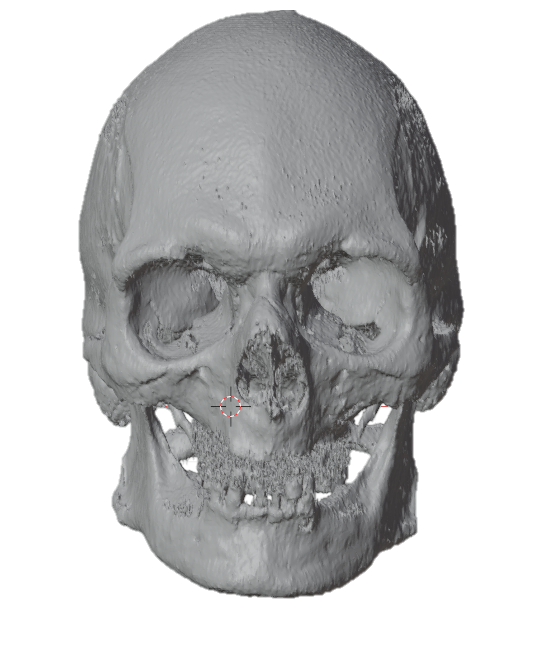


A

B

C

D


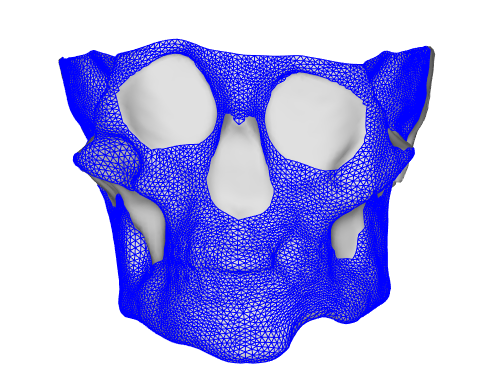

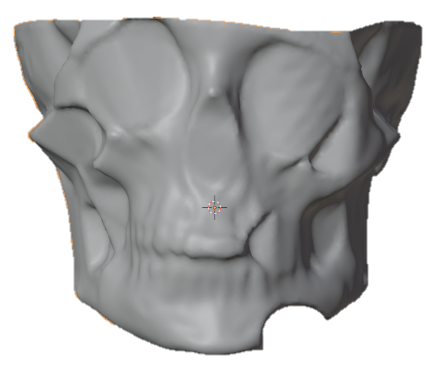

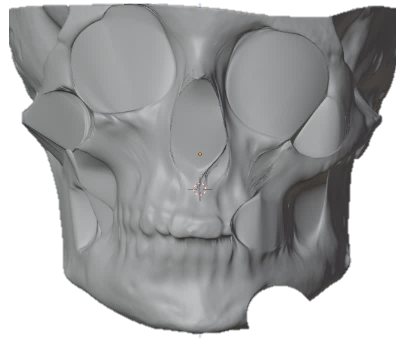

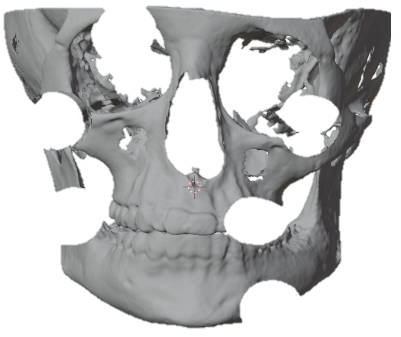

Supplement: Supplementary file 3 — Supplementary Information 3. [file 41598_2024_63137_MOESM3_ESM.docx]
